# Innovations in Digital Health From a Global Perspective: Proceedings of PRC‐HI 2024

**DOI:** 10.1002/hcs2.128

**Published:** 2025-01-24

**Authors:** Xiaoru Feng, Yu Sun, You Wu, Haibo Wang, Yang Wu

**Affiliations:** ^1^ School of Biomedical Engineering, Tsinghua Medicine Tsinghua University Beijing China; ^2^ School of Healthcare Management, Tsinghua Medicine Tsinghua University Beijing China; ^3^ Tsinghua University Press Beijing China; ^4^ School of Basic Medical Sciences, Tsinghua Medicine Tsinghua University Beijing China; ^5^ Department of Health Policy and Management, Bloomberg School of Public Health Johns Hopkins University Baltimore Maryland USA; ^6^ Research Centre of Big Data and Artificial Research for Medicine, The First Affiliated Hospital Sun Yat‐sen University Guangzhou China; ^7^ Department of Cardiovascular Surgery The First Medical Center of PLA General Hospital Beijing China

**Keywords:** digital health, medical artificial intelligence, robotics technology

**Abbreviations**
CT: computed tomography
EHRs: electronic health records
FAH‐SYSU: the First Affiliated Hospital, Sun Yat‐sen University
LLMs: large language models
PRC‐HI 2024: the Pacific‐Rim Conference on Healthcare Innovation

The rapid evolution of digital health technologies has sparked transformative changes across the healthcare landscape. These advancements were at the heart of discussions during the recent academic conference co‐organized by The First Affiliated Hospital, Sun Yat‐sen University (FAH‐SYSU) and University of California at Berkeley, the Pacific‐Rim Conference on Healthcare Innovation (PRC‐HI 2024), convening under the theme “The Future of Medicine: Integrating Robotics, AI and Healthcare.” This article distills the key developments and their implications for the future of healthcare, focusing on innovations in robotic surgery, health data science, and AI for medicine.

## 1 Transforming Surgical Robotics and Practices

Robotic surgery has become a cornerstone of modern surgical practices, offering enhanced precision, reduced recovery times, and lower complication rates. Dr. Xiaoyu Yin detailed advancements in robot‐assisted pancreatic surgeries at FAH‐SYSU, emphasizing the hospital's extensive experience with the Da Vinci surgical system. Since 2015, Dr. Yin has performed over 1000 robotic surgeries, including nearly 700 pancreatic resections. These procedures included advanced techniques such as robot‐assisted Whipple procedures, organ‐preserving pancreatectomies, and total pancreatectomies. His presentation highlighted the learning curves associated with these complex procedures, showcasing research on iterative improvements in surgical outcomes through case refinement and skill enhancement [1, 2].

Similarly, Dr. Junhang Luo presented a novel “gradual renal segmental artery off‐clamping” technique for treating large renal tumors. By utilizing preoperative computed tomography (CT) reconstructions, the technique identifies renal arterial branches, allowing surgeons to precisely minimize ischemia to healthy tissue while ensuring effective tumor removal. Clinical data revealed significantly shorter ischemia times, reduced blood loss, and improved long‐term renal function compared to traditional methods.

Dr. Qingbo Huang shared groundbreaking work on robotic telesurgery, particularly focusing on its applications in regions with limited medical resources. Through successful demonstrations of remote surgeries between Beijing and distant locations such as Sanya, Dr. Huang's research highlighted how low‐latency communication networks and advanced robotic systems can overcome geographical barriers [3].

Dr. Chao Cheng discussed the application of robotic surgery in thoracic procedures, particularly for lung cancer and large thymoma. His presentation highlighted how robotic systems enhance surgical precision and reduce recovery times, with notable success in segmentectomies and thymectomies [4]. The integration of 3D visualization and enhanced dexterity offered by robotic systems has transformed the management of challenging thoracic cases [5, 6].

Dr. Peter Nyirady presented on the potential of robotic surgery in addressing global surgical disparities. Highlighting the contributions of Semmelweis University, his team demonstrated how robotic systems have enhanced outcomes in urological surgeries. He also discussed future directions, such as semi‐autonomous surgical systems, and the importance of training programs to keep pace with these advancements.

Dr. Chris Fitzpatrick elaborated on the use of AI and data analytics in optimizing robotic surgical practices. Through platforms such as Case Insights, his research highlighted how analyzing surgical video data and performance metrics can identify skill gaps and provide actionable feedback for surgeons [7, 8, 9, 10]. This approach has the potential to standardize training and improve surgical outcomes globally.

## 2 Robotics Beyond Surgery: Assistive and Social Applications

Dr. Veronica Ahumada‐Newhart introduced the potential of telepresence robots in improving social inclusion for children with mobility impairments. Her studies revealed that these robots enable remote participation in classroom activities, such as attending lessons and engaging in physical education, reducing feelings of isolation and fostering a sense of community [11, 12]. However, technical challenges, such as screen visibility and interaction limitations due to robot design, remain significant for children. As these technologies advance, their applications could extend beyond pediatrics, supporting elderly care and mental health interventions.

## 3 Digital Health Frontiers: Precision Medicine and Beyond

Dr. Martin Loos highlighted the integration of big data analytics into postoperative management strategies for pancreatic surgeries. By leveraging detailed surgical data from Heidelberg, his research team has been able to identify key factors influencing patient outcomes across different types of total pancreatectomy [13].

Dr. Brad Pollock highlighted data‐driven technologies in public health, focusing on applications in health protection, health promotion, and healthcare delivery. Technologies like wastewater monitoring, geospatial analysis, and exposomics improved COVID‐19 disease surveillance [14]. For health promotion, approaches such as serious gaming technology increased treatment adherence [15] while newer generative AI and social media analytics will enhance behavioral interventions. Data‐driven technology will continue to improve diagnostic screening and optimize health resource allocation.

Dr. Michael Wang delved into the historical and current trends of digital healthcare, categorizing its evolution into three phases: Digital Medicine 1.0, focused on digitizing healthcare systems; Digital Medicine 2.0, emphasizing data‐driven insights; and Digital Medicine 3.0, which integrates advanced AI models for predictive and precision medicine. He also emphasized the ongoing challenges of data security and interoperability.

Dr. Ruchi Thanawala introduced “Epistomics,” an innovative approach that studies how learning occurs within medical education and practice. She discussed how big data from robotic surgery—such as videos, kinematics, and patient outcome data—combined with an interdisciplinary learning framework, can enhance educational outcomes and promote equity in training.

In addition, Dr. Frederick P. Ognibene emphasized the importance of effective teamwork and mentorship in advancing digital health research. He believed that clear timelines, role assignments, and open communication are key to ensuring research success. Successful teamwork relies on trust, communication, and shared scientific goals.

## 4 AI‐Powered Tools and Their Impact on Healthcare

Dr. Haibo Wang provided a comprehensive overview of the development status and challenges of medical AI in China. While acknowledging China's competitive edge in research achievements and international collaborations, he highlighted gaps in patent approvals, technology transfer, and competition in high‐end international markets. He also delved into the three core thinking pathways of AI and their critical roles in optimizing diagnostic and treatment processes [16, 17, 18], highlighting the shift of medical AI from task‐specific to universal health‐centered [19]. Additionally, he emphasized major challenges such as data scarcity, model failure risks, and ethical considerations in AI applications [20].

Dr. Joseph Sung explored the evolving role of physicians in an era increasingly influenced by AI and robotics. He provided examples from gastroenterology, including AI‐powered colonoscopy tools that improve detection rates for abnormalities and reduce colorectal cancer mortality [21]. Despite the promise of AI, Dr. Sung emphasized that critical decision‐making and patient interaction remain the core responsibilities of clinicians, advocating for AI as an augmentative rather than a replacement tool in healthcare [22].

Dr. Katherine Kim highlighted the emerging role of digital twin technology in healthcare, focusing on its applications in personalized medicine, disease prediction, and care management [23]. She proposed a healthcare digital twin framework encompassing five key areas: purpose, application levels, multimodal data sources, model types, and methods and technologies.

Dr. Yang Liu explored iFlytek's AI‐driven solutions for primary care in China. Leveraging over 3 billion medical records, iFlytek's Smart Medical Assistant system has revolutionized chronic disease management by supporting patients through the entire care continuum—from pre‐hospital health screenings to in‐hospital disease adherence and medication adjustments, and post‐discharge health monitoring with personalized treatment recommendations.

Dr. Nicholas Anderson reviewed the evolution of electronic health records, from initial digital transformation to the adoption of deterministic AI, and the emergence of generative AI. Generative AI demonstrates immense potential in handling various data types, automating documentation processes, and generating new data, offering promising prospects for optimizing clinical decision‐making and improving workflow efficiency [24, 25].

Dr. Weizhi Ma presented the concept of “agent hospitals” utilizing generative AI and large language models to create virtual medical systems that drive innovation in medical education and clinical practice. By simulating interactions between patients and doctors, AI doctors engage in self‐learning and evolution within the virtual environment, covering 21 specialties and generating hundreds of thousands of virtual cases to support medical education and medical decisions [26].

Dr. Jingwen Zhang explored how AI is transforming communication in healthcare, highlighting its potential to improve diagnostic accuracy and reduce gender and racial biases [27, 28, 29]. However, she also noted the challenges AI faces in building trust with patients and balancing professionalism with empathy, calling for attention to AI's ethics, safety, privacy, and fairness to promote more efficient and equitable health communication and healthcare development [30, 31].

## 5 Conclusion

The conference provided a comprehensive overview of the transformative impact of digital health innovations. From enhancing medical education to addressing disparities in care delivery, the discussions highlighted both the opportunities and challenges of integrating these technologies into practice. The detailed scientific research presented underscores the potential for interdisciplinary collaboration and ethical governance to maximize the benefits of digital health. Readers are encouraged to explore the references provided for deeper insights into these groundbreaking advancements.

## Author Contributions

**Xiaoru Feng:** writing–original draft (equal). **Sun Yu:** resources (equal). **You Wu:** conceptualization (equal), writing–review and editing (equal). **Haibo Wang:** conceptualization (equal), resources (equal). **Yang Wu:** supervision (equal), writing–review and editing (equal).

## Ethics Statement

The authors have nothing to report.

## Consent

The authors have nothing to report.

## Conflicts of Interest

Professor Haibo Wang is a member of the *Health Care Science* Editorial Board. To minimize bias, he was excluded from all editorial decision‐making related to the acceptance of this article for publication. The remaining authors declare no conflicts of interest. Yu Sun is the Editorial Office Director of *Health Care Science* and is not involved in all the editorial decision related to the publication of this article. This article belongs to a special issue (SI)‐*Implementation, Innovations and Challenges of Digital Health*. As the journal's Editorial Board Member and SI's Guest Editor, Professor Yang Wu is excluded from all the editorial decision related to the publication of this article.

## Data Availability

The authors have nothing to report.
